# Development of UNU-Casemix Grouper standard integration tool kit for hospital information system (HIS) in selected hospitals in Malaysia and Indonesia

**DOI:** 10.1186/1471-2458-14-S1-P17

**Published:** 2014-01-29

**Authors:** Syed Mohamad Hamzah Al-Junid, Syed Mohamed Aljunid, Zafar Ahmed, Hasrul Reeza Mustaffa

**Affiliations:** 1 United Nations University-International Institute for Global Health, UKMMC Complex, Jalan Yaacob Latiff, Bandar Tun Razak, 56000 Cheras, Kuala Lumpur, Malaysia; 2 Jabatan Kesihatan Masyarakat, Pusat Perubatan Universiti Kebangsaan Malaysia, Jalan Yaacob Latif, Bandar Tun Razak, 56000 Cheras, Kuala Lumpur, Malaysia

## Background

UNU-Casemix Grouper is software used for grouping inpatient and outpatient cases into Case Base Group (CBG). UNU-Casemix can be used to enhance quality and efficiency of healthcare services. Hospital Information System (HIS) is used to collect all the patient-level data in a hospital that can be used to improve performance of a hospital. HIS also captures all of the patient’s demographic and clinical data required by the UNU-Casemix Grouper. Combining both systems in one platform can ensure that hospitals gain benefit from both systems. The main objective of the study was to develop a standard integration tool kit for UNU-Casemix Grouper for selected HIS hospitals in Malaysia and Indonesia.

## Materials and methods

In this study, a General System Theory (GST) will be used to assess the current HIS used in the hospitals in terms of database, platform, coding language and the Casemix data available in the HIS. This information will be used in the Rapid Application Development (RAD) method as an input for developing a standard tool for UNU-Casemix Grouper integration tool kit. RAD is a method base on Software Development – Life Cycle (SDLC) that uses minimal planning in favour of rapid prototyping. RAD does not require extensive pre-planning that allows software to be written much faster, and easier to be modified according to requirements. As a result, the required data for the CBG development will be extracted from HIS database, formatted and processed by the UNU-Casemix Grouper.

## Conclusions

With the integration tool UNU-Casemix grouper can be embedded within the HIS of selected hospitals. HIS data collected in the hospital can be used to develop the Casemix system in the hospitals in a seamless manner.

